# Citalopram intoxication in four week old infant

**DOI:** 10.1186/s12887-020-02439-5

**Published:** 2020-12-07

**Authors:** Jo-Anne Janson, Arthur T. M. Wasylewicz, Marianne Eijkemans, Marieke Kerskes

**Affiliations:** 1 Pediatrics, Maastricht UMC+, Maastricht, The Netherlands; 2 Pharmacology, Catharina Hospital, Eindhoven, The Netherlands; 3 Pediatrics, Catharina Hospital, Michelangelolaan 2, 5623 EJ Eindhoven, The Netherlands

**Keywords:** Citalopram, SSRI, Infant, Intoxication, Finnegan scores

## Abstract

**Background:**

In contrast to intoxications in toddlers which can be due to accidental ingestions, many intoxications in infants are due to medication errors**.** To our knowledge, this is the first case report of a citalopram intoxication in an infant, and may offer new insight on possible screening methods for intoxication as well as pharmacokinetics of citalopram in small infants.

**Case presentation:**

This case report describes an unintentional citalopram intoxication in a 4 week old infant due to a vitamin D drops ‘look alike’ error. The infant showed extreme jitteriness and opisthotonus at presentation, as well as prolonged signs of gastro-oesophageal reflux. No cardiac rhythm disturbances or convulsions were seen. The clinical course combined with Finnegan scores was correlated to and supported by pharmacokinetic and pharmacokinetic data of citalopram in the patient.

**Conclusions:**

Using Finnegan scores in general pediatric practice could help objectify follow-up of acute intoxications in young infants with neurological symptoms.

**Supplementary Information:**

The online version contains supplementary material available at 10.1186/s12887-020-02439-5.

## Background

Citalopram is the second most commonly used antidepressant in the world [1]. Citalopram is used in treating depression in pregnant as well as postpartum women, and is the most used antidepressant in pregnancy [1–3]. Citalopram is a selective serotonin reuptake inhibitor (SSRI), it works by inhibiting the central nervous system (CNS) reuptake of serotonin (5-HT) and the potentiation of serotonergic activity. Citalopram intoxication in adults is usually mild, consisting of symptoms of nausea, dizziness, tremors and somnolence, nevertheless significant toxicity can occur [4]. In children however, it has been defined as the most hazardous intoxication of all SSRIs, with seizures occurring four times more frequent that other SSRI’s, as well as cardiotoxic effects, which occur more than three times as much [5].

In contrast to intoxications in toddlers which are often due to accidental ingestions, many intoxications in infants are due to medication errors [6]. Common medication errors which can occur in a ‘home’ situation are often due to ‘look-alike, sound-alike’ drug names and packaging [7]. In this case report, we describe an infant that was given his mother’s citalopram drops instead of its vitamin D drops, which are recommended to prevent rickets [8]. Little is known about the toxicity or kinetics of citalopram in infants [5]. To our knowledge, this is first case report of a citalopram intoxication in an infant, and may offer new insight on possible screening methods for intoxication as well as some insight into the pharmacokinetics of citalopram in small infants.

## Case presentation

**Fig. 1:**
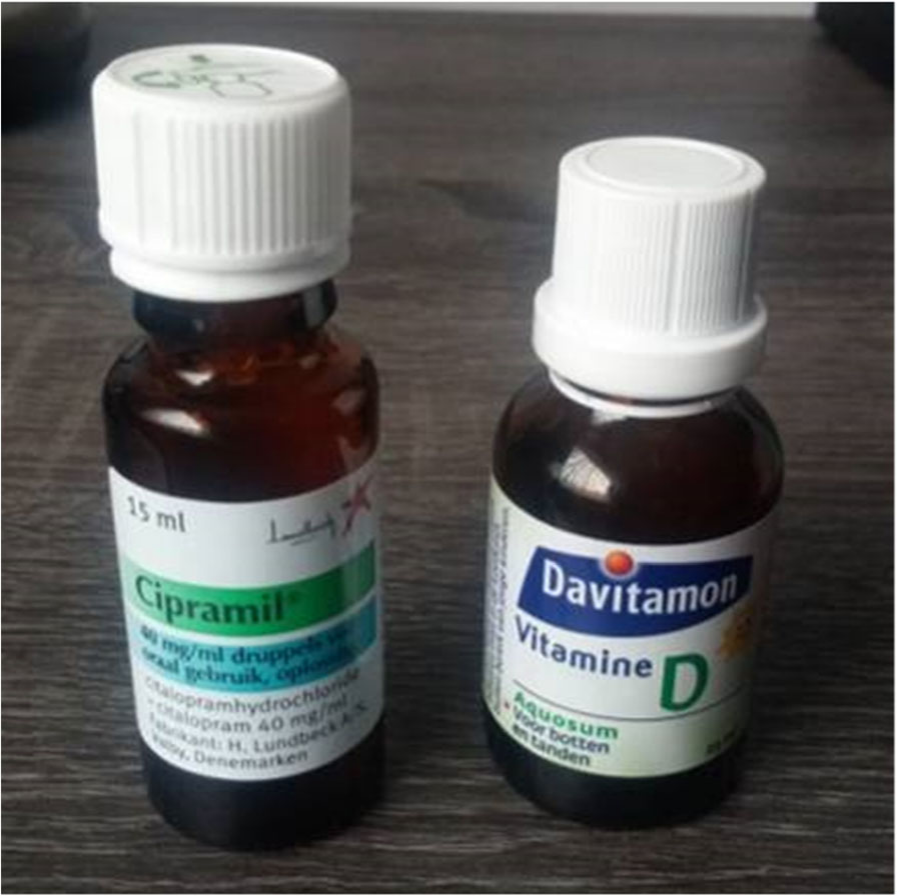
Photo showing the citalopram (left) and vitamin D (right). (Picture provided by the parents)

A previously healthy, 4 week old, Caucasian boy was brought to the emergency department by his mother because he had accidentally been given the mothers’ dose of citalopram. Instead of the vitamin D drops she intended to give him, she accidentally switched bottles of the citalopram and vitamin D. These bottles are similar, as shown in Fig. 1. The mother noticed the incorrect medication bottle around 30 min later and immediately presented the infant to the emergency department. She administered 10 drops of citalopram, corresponding to 20 mg. With a weight of 3355 g corresponding to a dose of around 6.0 mg/kg.

**Fig. 2:**
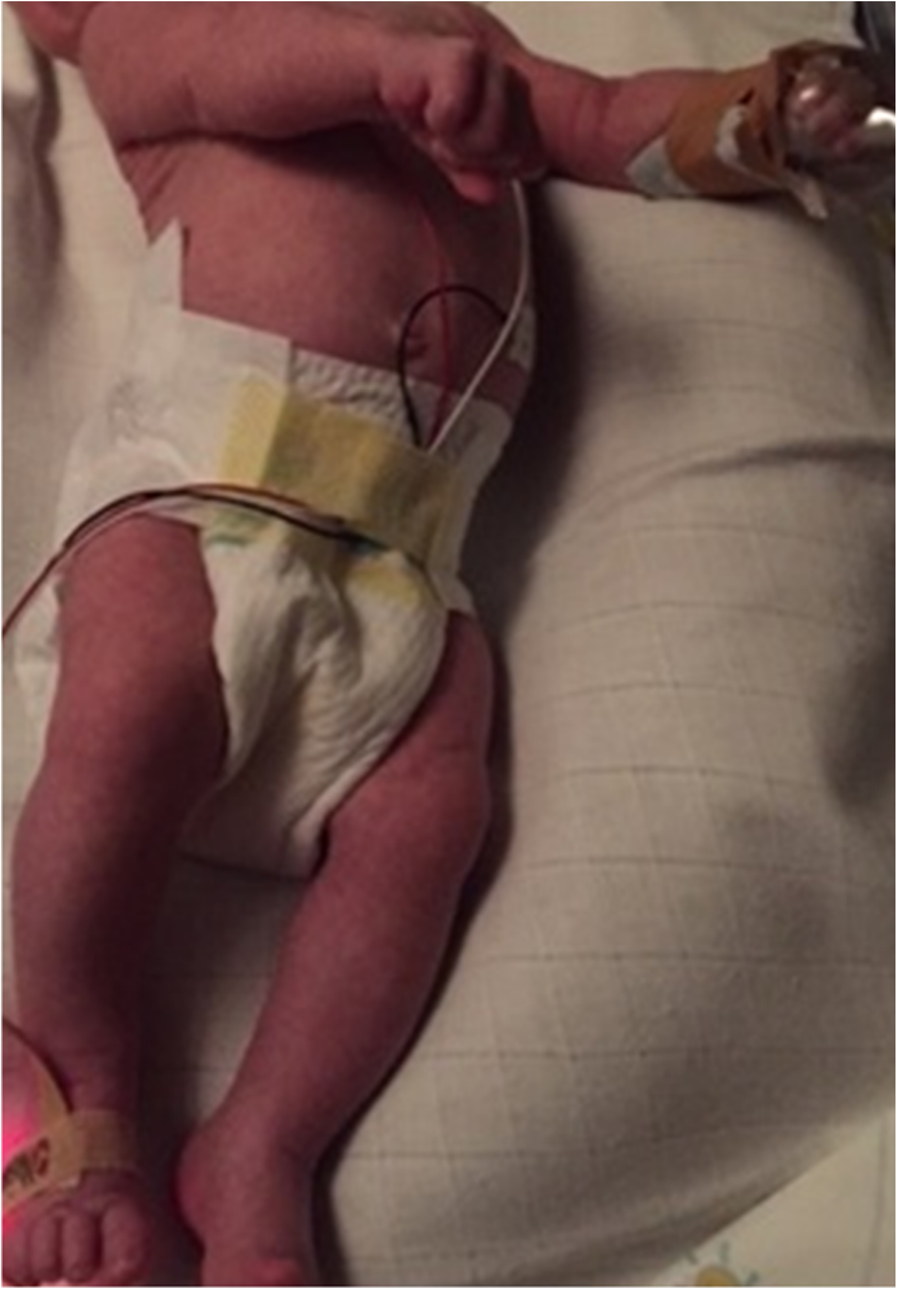
The infant at presentation at the emergency department showing clear signs of opisthotonos (placed with permission of the parents)

Upon first physical examination we saw an extremely jittery, agitated infant with an evident opisthotonos, as shown in Fig. 2. Vital signs were stable, the patient had a respiratory rate of 50/min and an oxygen saturation of 100% in room air. At presentation the patient showed an isolated systolic hypertension. Blood pressure was 110/38 mmHg (reference value p95 for systolic pressure 105 mmHg), with a regular heart rate of 190 beats per minute [9]. An electrocardiogram showed sinus tachycardia with normal intervals. A nasogastric tube was inserted to empty the stomach; activated charcoal and sodium sulfate were administered over a 2 h period to prevent any possible further absorption of the citalopram. Furthermore, an intravenous cannula was placed with maintenance fluid and secure intravenous access.

The patient was transferred to a neonatal intensive care unit (NICU) for intensive monitoring, concerning the possible risk of cardiac rhythm disturbances and convulsions. Laboratory testing was performed including a complete blood count, infection parameters and electrolyte concentration, all results were within normal limits. Because of the risk of convulsions and intracerebral hemorrhage, amplitude-integrated electroencephalography (aEEG) and cerebral ultrasound were performed, which showed no abnormalities.

After 16 h the patient was transferred back to the medium care neonatal ward. In the following days he continued showing signs of extreme jitteriness and increased muscle tone. After 4 days these symptoms were found to be acceptable enough to discharge the patient. Before, during and after admission the patient was only fed formula feeding, breast milk was not used.

During several weeks, at ambulant follow-up, the patient showed persistent signs of increased muscle tone for at least 1 month after discharge. Furthermore, he showed signs of agitation and gastro-oesophageal reflux. Treatment was started with esomeprazole to counteract the effect of the gastro-oesophageal reflux. After 7 months patients symptoms disappeared completely and the patient was discharged from further ambulant follow-up.

### Pharmacokinetic and pharmacodynamic evaluation

To assess pharmacokinetic parameters as well as to predict time to improvement of symptoms, citalopram and the active metabolite desmethylcitalopram concentrations were measured in the serum of the patient. Citalopram and desmethylcitalopram concentrations were analyzed using a validated UPLC-MS-MS method at the Onze Lieve Vrouwe Gasthuis hospital in Amsterdam, the Netherlands. Two hours after ingestion, the citalopram plasma concentration was 77 μg/l with no detectable desmethylcitalopram concentration. Fifty-four hours after ingestion, citalopram serum level decreased to 33 μg/l and desmethylcitalopram increased to a concentration of 43 μg/l.

**Fig. 3:**
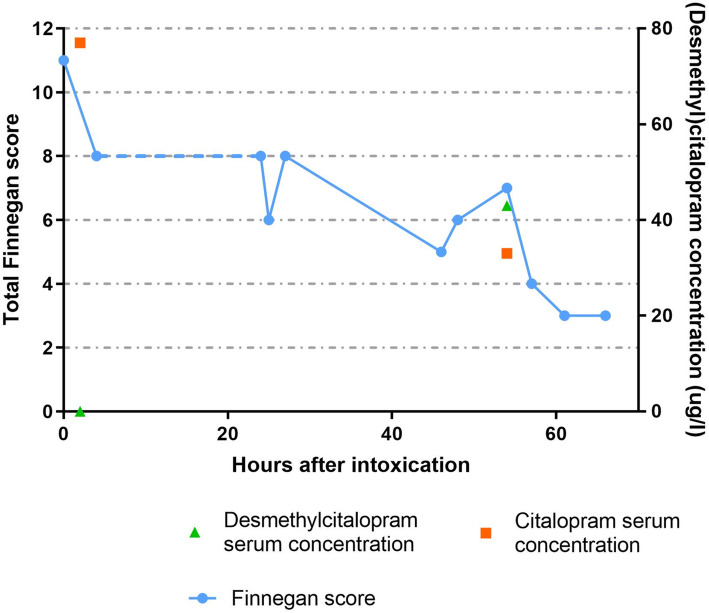
Pharmacokinetics and pharmacodynamic parameters after ingestion of 20 mg citalopram by a 4 week old infant. Legend: Figure shows Finnegan scores throughout admission taken at random times. The blue dots represent the Finneganscores. Dotted blue line represents NICU admission where no Finneganscores were recorded. Scoring is displayed on the primary Y-axis on the left side of the figure. The orange dots represent the citalopram serum levels and the green dots the desmethylcitalopram serum levels, concentration is displayed on the secondary Y-axis on the right side of the figure

To objectify the severity and clinical course, Finnegan scores were randomly measured during the course of admission (Additional file 1) [10]. Fig. 3 shows the course of the known Finnegan scores, citalopram and desmethylcitalopram serum levels. Finnegan scores were as follows: 2 h after ingestion 11, 24 h after ingestion 8, 46 h after ingestion 6, 66 h after ingestion 3, more elaborate data is shown in Additional file 1. Unfortunately during the time of admittance at the NICU, Finnegan scores were not measured.

## Discussion and conclusions

In this case report we presented a 4 week old infant which was accidently given 20 mg citalopram resulting in a dose of 6.0 mg/kg, being around 30 times a ‘recommended’ dose in older children. The most prominent symptoms were an opisthotonos and persistent jitteriness.

This case report describes an unintentional citalopram intoxication due to a vitamin D drops ‘look alike’ error. It is of great importance that clinicians are aware of the fact that packaging of drops can be similar. In this case, a warning was sent to the central medication incident database, to prevent further mix-ups.

At presentation 2 h after ingestion, the serum citalopram concentration was 77 μg/l, much lower than the reported toxicity levels in adults, starting at 400 μg/l. [4] However, the measured concentration might not yet be the peak concentration, as the Tmax is about 2 h for Cipramil® drops [11, 12]. In a similar case report, a 10 month old infant ingested an unknown amount of citalopram tablets, the initial plasma level (1 h after ingestion) was 1400 μg/l. [13] In contrast to our case, this infant developed seizures requiring anti-epileptic therapy, making it impossible to administer repeated doses of activated charcoal and laxative.

While neurological symptoms dissipated during admission, the patient kept on showing signs of increased muscle tone and gastro-oesophageal reflux during several weeks. A possible explanation for these symptoms could be high levels of the active metabolite desmethylcitalopram [14]. Around 80% of citalopram is metabolized via hepatic cytochrome P450 system, CYP2C19, CYP3A4 and CYP2D6 playing a major role. It is metabolized, into several metabolites of which only desmethylcitalopram is known to be pharmacologically active. The active metabolite desmethylcitalopram has a longer half-life than citalopram being around 50 h in adults [15] and possible playing a major role in the therapeutic effect of the treatment [16]. However, no citalopram and desmethylcitalopram plasma levels were measured at follow-up to support this hypothesis.

Because there are no scores to objectify seriousness and clinical course of intoxications in infants we chose to use the Finnegan scores (Additional file 1). The Finnegan score is an objective method used to monitor neonatal abstinence symptoms. A score of eight or higher in three measurements indicates the need for therapy. Finnegan scores measure: high pitched cries, sleep-disturbances, hyperactive reflexes, tremors, convulsions, hyperthermia, sneezing, tachydyspnea and feeding problems. Previous studies show values above eight can be considered pathological in a 4 week old infant, and can be interpreted as a sign of narcotic withdrawal [17]. While the Finnegan scores seemed to correlate to neurological course during admission, it did not adequately correlate to the gastro-intestinal symptoms that persisted like the GER and increased muscle tone. A possible explanation is that Finnegan scores mainly subjectively quantify the level of neurologic excitability, and in lesser amount gastrointestinal dysfunction [18].

We suggest using the Finnegan scores in future SSRI intoxications in infants to objectify and predict acute clinical course. Keep in mind that the Finnegan-scores can be only be used up until 4 weeks of age [10]. The symptoms that occurred could be labeled as severe serotonin syndrome, where the Hunter criteria can be useful for diagnosis [19]. We did not use these criteria during the clinical course. Further research is needed because the Hunter criteria are not validated for the pediatric population. We describe a unique case-report concerning a 4 week old infant with accidental citalopram intoxication. The patient presented with extreme jitteriness and increased muscle tone. Fortunately, no serious adverse effects were monitored. During the clinical course, we have shown Finnegan scores can be helpful in determining toxicity and half-life of citalopram.

The clinical course seems to be correlated to the Finnegan scores, this seems partly supported by pharmacokinetic data of citalopram in a young infant, previously not described in literature. We believe that using Finnegan scores in general pediatric practice could help objectify follow-up of intoxications in young infants with neurological symptoms. Moreover, by presenting this case we hope to invite researchers to validate Finnegan scores to use in intoxications of young infants.

## Supplementary Information

**Additional file 1.** Finnegan scores of patient.

## Data Availability

The datasets used and/or analysed during the current study are available from the corresponding author on reasonable request.

## Abbreviations

SSRI: Selective Serotonin Reuptake Inhibitor
CNS: Central nervous system
5-HT: Serotonin
NICU: Neonatal intensive care unit
UPLC-MS-MS: Ultra-performance liquid chromatography-tandem mass spectrometry
Cmax: Maximum (or peak) serum concentration
T ½: Drug elimination half-life
