# Supplementary material for: Citalopram intoxication in four week old infant
Source: BMC Pediatr. 2020 Dec 7;20:552. doi: 10.1186/s12887-020-02439-5 (PMC7722423; doi:10.1186/s12887-020-02439-5)
Supplement: Supplementary file 1 — Additional file 1. Finnegan scores of patient. [file 12887_2020_2439_MOESM1_ESM.docx]

**Additional files**

**Additional file 1 :** Finneganscores of patient

| Time after ingestion (h) | Finnegan score | Sk | S: | T: | Musc | Temp | RR: | GI: | Citalopram serum levels  (ug/L) | Desmethyl-citalopram (ug/L) |
| --- | --- | --- | --- | --- | --- | --- | --- | --- | --- | --- |
| 2 | 11 |  |  |  |  |  |  |  | 77 |  |
| **NICU MMC** |  |  |  |  |  |  |  |  |  |  |
| 24 | 8 | 1 | - | 3 | 2 | 1 | 1 | - |  |  |
| 25 | 6 | 1 | - | 2 | 2 | 1 | - | - |  |  |
| 27 | 8 | 1 | 2 | 1 | 2 | 1 | 1 | - |  |  |
| 46 | 5 | 1 | - | 3 | - | 1 | - | - |  |  |
| 48 | 6 | 1 | 1 | 3 | - | 1 | - | - |  |  |
| 52 | 6 | 1 | 1 | - | - | 1 | - | 2 |  |  |
| 54 | 7 | 1 | 2 | 1 | - | 1 | - | 2 | 33 | 43 |
| 57 | 4 | 1 | 2 | - | - | - | 1 | - |  |  |
| 61 | 3 | 1 | - | - | 2 | - | - | - |  |  |
| 66 | 3 | 1 | - | - | 2 | - | - | - |  |  |
| Sk: Skin, S: Sleep, T: tremors, Musc: Muscles, Temp: Temperature, RR: Respiratory rate, GI: GI tract | | | | | | | | | | |
